# Better together: Elements of successful scientific software development in a distributed collaborative community

**DOI:** 10.1371/journal.pcbi.1007507

**Published:** 2020-05-04

**Authors:** Julia Koehler Leman, Brian D. Weitzner, P. Douglas Renfrew, Steven M. Lewis, Rocco Moretti, Andrew M. Watkins, Vikram Khipple Mulligan, Sergey Lyskov, Jared Adolf-Bryfogle, Jason W. Labonte, Justyna Krys, RosettaCommons Consortium, Christopher Bystroff, William Schief, Dominik Gront, Ora Schueler-Furman, David Baker, Philip Bradley, Roland Dunbrack, Tanja Kortemme, Andrew Leaver-Fay, Charlie E. M. Strauss, Jens Meiler, Brian Kuhlman, Jeffrey J. Gray, Richard Bonneau

**Affiliations:** 1 Center for Computational Biology, Flatiron Institute, Simons Foundation, New York, NY, United States of America; 2 Dept of Biology, New York University, New York, NY, United States of America; 3 Dept of Chemical and Biomolecular Engineering, Johns Hopkins University, Baltimore, MD, United States of America; 4 Dept of Biochemistry, University of Washington, Seattle, WA, United States of America; 5 Institute for Protein Design, University of Washington, Seattle, WA, United States of America; 6 Lyell Immunopharma, Seattle, WA, United States of America; 7 Dept of Biochemistry and Biophysics, University of North Carolina at Chapel Hill, Chapel Hill, NC, United States of America; 8 Dept of Biochemistry, Duke University, Durham, NC, United States of America; 9 Cyrus Biotechnology, Seattle, WA United States of America; 10 Dept of Chemistry, Vanderbilt University, Nashville, TN, United States of America; 11 Dept of Biochemistry, Stanford University School of Medicine, Stanford CA, United States of America; 12 Dept of Immunology and Microbiology, The Scripps Research Institute, La Jolla, CA, United States of America; 13 Dept of Chemistry, Franklin & Marshall College, Lancaster, PA, United States of America; 14 Dept of Chemistry, University of Warsaw, Warsaw, Poland; 15 Dept of Biological Sciences, Rensselaer Polytechnic Institute, Troy, NY, United States of America; 16 Dept of Microbiology and Molecular Genetics, IMRIC, Ein Kerem Faculty of Medicine, Hebrew University of Jerusalem, Jerusalem, Israel; 17 Fred Hutchinson Cancer Research Center, Seattle, WA, United States of America; 18 Institute for Cancer Research, Fox Chase Cancer Center, Philadelphia PA, United States of America; 19 Dept of Bioengineering and Therapeutic Sciences, University of California San Francisco, CA, United States of America; 20 Bioscience Division, Los Alamos National Laboratory, Los Alamos, NM, United States of America; 21 Depts of Chemistry, Pharmacology and Biomedical Informatics, Vanderbilt University, Nashville, TN, United States of America; 22 Center for Structural Biology, Vanderbilt University, Nashville, TN, United States of America; 23 Institute for Chemical Biology, Vanderbilt University, Nashville, TN, United States of America; 24 Institute for Drug Discovery, Leipzig University, Leipzig, Germany; 25 Dept of Computer Science, New York University, New York, NY, United States of America; 26 Center for Data Science, New York University, New York, NY, United States of America; Hebrew University of Jerusalem, ISRAEL

## Abstract

Many scientific disciplines rely on computational methods for data analysis, model generation, and prediction. Implementing these methods is often accomplished by researchers with domain expertise but without formal training in software engineering or computer science. This arrangement has led to underappreciation of sustainability and maintainability of scientific software tools developed in academic environments. Some software tools have avoided this fate, including the scientific library Rosetta. We use this software and its community as a case study to show how modern software development can be accomplished successfully, irrespective of subject area. Rosetta is one of the largest software suites for macromolecular modeling, with 3.1 million lines of code and many state-of-the-art applications. Since the mid 1990s, the software has been developed collaboratively by the RosettaCommons, a community of academics from over 60 institutions worldwide with diverse backgrounds including chemistry, biology, physiology, physics, engineering, mathematics, and computer science. Developing this software suite has provided us with more than two decades of experience in how to effectively develop advanced scientific software in a global community with hundreds of contributors. Here we illustrate the functioning of this development community by addressing technical aspects (like version control, testing, and maintenance), community-building strategies, diversity efforts, software dissemination, and user support. We demonstrate how modern computational research can thrive in a distributed collaborative community. The practices described here are independent of subject area and can be readily adopted by other software development communities.

This is a *PLOS Computational Biology* Software paper.

## Introduction

Tackling grand challenges in various scientific disciplines requires ideas and perspectives from team members covering a diverse range of expertise, with sustained focus that persists over a time frame sufficiently long to meet bold scientific objectives[1,2]. But how do you assemble a team that can successfully work together on a shared goal over long periods of time? A recent National Academies report[1] summarized key challenges for “team science” as including diversity of membership, interdisciplinary knowledge integration, large size, goal alignment, geographic dispersion, and task interdependence[1]. We share here how our team has met these challenges. Our collective experience in the RosettaCommons consortium has been marked by extraordinary collaboration in a team composed of smaller groups that each innovate on their own projects. This teamwork is possible because our work centers around a shared software suite. The rise of shared, distributed scientific software tools across many scientific disciplines presents an opportunity for forming focused, productive research communities. In this paper, we share the key technical, social, and dissemination practices that have enabled successful scientific software development in our collaboration, the RosettaCommons[3]. The paper complements two recent, detailed, technical reviews about Rosetta that discuss latest applications[4] and the state and history of the score function[5].

**Fig 1:**
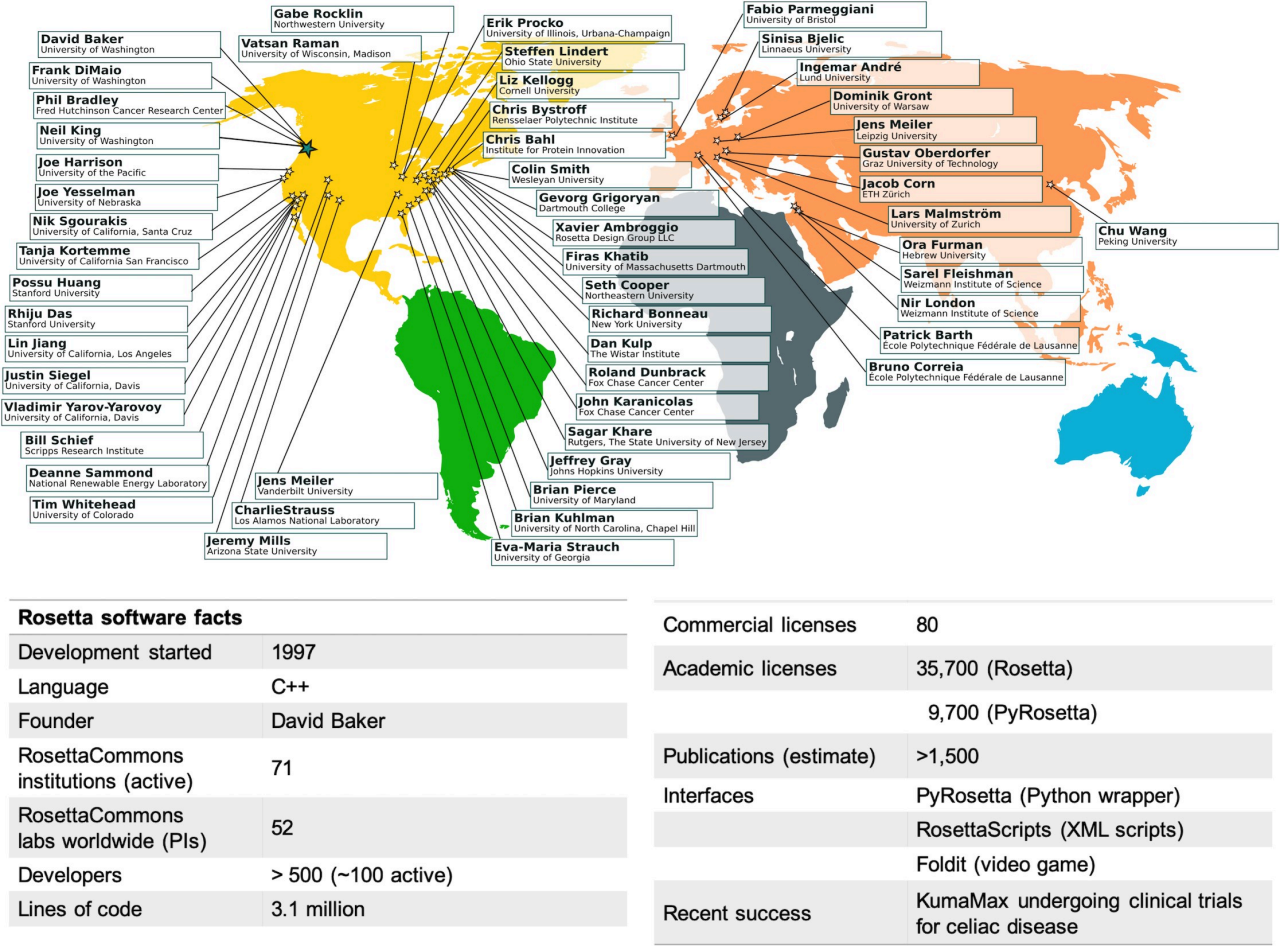
RosettaCommons facts. Main laboratories and institutions in the RosettaCommons and basic facts about our software in 2019.

Computational skills are increasingly vital across a broad range of scientific disciplines. However, few training programs in these fields include instruction in computer science[6] or software engineering. Thus, many scientists implement their workflows in transient scripts or programs focused only on addressing an immediate concern. These programs often lack generality, extensibility, code comments, or documentation, and can be difficult to integrate with other methods[7]. The general lack of documentation or example workflows (“protocol captures”[8,9]) further diminishes reproducibility and usability[10,11]. In contrast, accomplishing complex tasks and pushing scientific boundaries generally requires reuse of sophisticated, validated algorithms. Appropriately generalized, shared code can allow new, complex methods to be built from simpler, reusable modules that combine ideas from multiple sources. We have used such a model to develop the macromolecular modeling software suite Rosetta[4] (Fig 1). The project’s success and longevity have relied on the cultivation of a community around the codebase with the larger goal of solving complex scientific problems by sharing ideas and tools and collaborating closely.

In addition to solving technical problems for scientific advancements, our community established coding, publication, and community-interaction standards that enabled continued growth and health of the software and our community over many years. We meet at least twice a year to discuss broad changes to the codebase and to share our newest tools and the scientific advances made with them. Additionally, we form new collaborations at these meetings and recognize members who have made important contributions to the codebase and/or the community. To ensure rapid entry into development, we organize regular training sessions for newcomers across the community. We created a code of conduct, which we require our members to adhere to, regardless of their institutions. We are closely connected to our extensive user base to further improve our software and apply it to real-world scientific problems. Our community has changed the way we work, especially in conjunction with relatively recent tools that allow social interaction on various levels (GitHub, Slack, video conferences).

**Fig 2:**
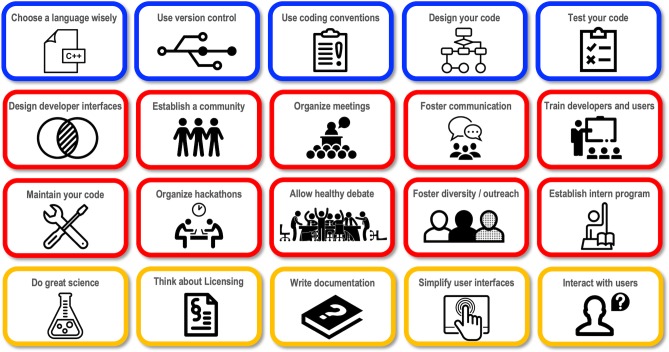
Pillars of success in the development of scientific software. Technical aspects are in blue, social aspects in red and dissemination and user interaction in yellow. This figure was created with icons from The Noun Project[13].

Here we describe the social and technical elements that have shaped our software[4,12] and community over the past two decades, including mistakes and lessons learned. We focus on three major categories: technical aspects (*e*.*g*. version control and testing frameworks), social cohesion (*e*.*g*. meetings, communication, inclusion and outreach), and software dissemination (*e*.*g*. licensing, documentation, and user interaction). We believe that many of these features (Fig 2) can be translated to other communities. Thus, we hope this review can help other groups grapple with the many questions in performing collaborative research, from implementing effective software engineering practices to balancing healthy competition with effective use of shared resources, to inclusion of diverse groups of people and practicing transparent and inclusive decision-making strategies.

### Beginnings and growth of our community

**Fig 3:**
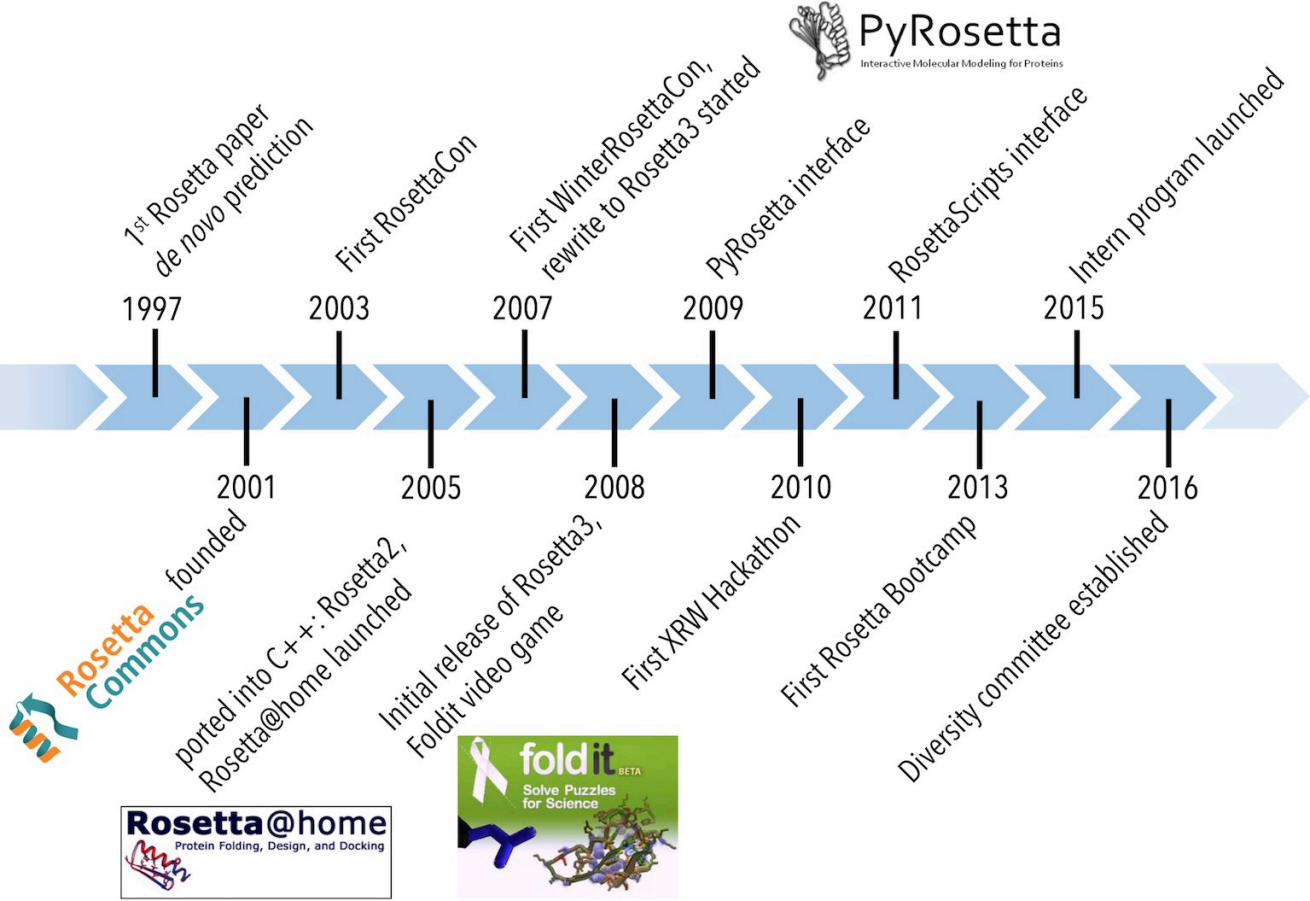
Rosetta history. Major milestones in the history of Rosetta development.

Development of our software suite started in the mid 1990s in David Baker’s laboratory at the University of Washington (Fig 3). Rosetta was originally developed for protein structure prediction and to gain insights into protein folding, which remains a grand challenge in theoretical biophysics and underlies our understanding of biology, human health and disease. The project started when two graduate students implemented a Monte Carlo sampling algorithm[14] and a scoring function[15] that consisted of physics-based and knowledge-based terms. The protocol was successful in the blind prediction challenges, CASP 3[16] and 4[17]. Following this success, several postdoctoral fellows joined the project to develop various structure prediction and design applications.

As the early developers began independent positions at various institutions, they continued developing and improving their modeling applications, and it became clear that enhancing collaboration across institutions would be beneficial to scientific progress. This led to the creation of the RosettaCommons community of developers, which involved a unique intellectual property agreement, described in the licensing section below. The RosettaCommons structure allowed seamless collaboration across many institutions, which spurred developments in many different directions. By 2019, the RosettaCommons has grown to laboratories at 71 institutions worldwide, overseeing a project consisting of over 3 million lines of code with contributions from over 800 scientists. The code has been licensed to ~35,700 academic scientists and 80 commercial entities.

## Technical aspects

### Language history of the codebase

The early codebase was written in Fortran and later machine-translated into C++ (by Objexx Engineering), which was released in the summer of 2005 as Rosetta++ (also known as Rosetta2). Over the next two years, the automatic translation was found to be unworkable for continued development, and a software engineer was hired to implement the software as an object-oriented library, called *librosetta*. Although the first attempt at creating an object-oriented hierarchy turned out to be unwieldy and inflexible and ultimately had to be abandoned, it gave us insight into better choices for our central objects and their relationships. The next object-oriented rewrite of the codebase was initiated in the summer of 2007 by two of our core developers who were both scientists and software engineers (Andrew Leaver-Fay, a member of the Kuhlman lab, and Phil Bradley, at the Fred Hutchinson Cancer Research Center). This code was initially called *miniRosetta* and would later become Rosetta3, released in early 2009[12]. *MiniRosetta* also remedied a design flaw of Rosetta++ which presented as a single monolithic application with input options that allowed many different protocols to be invoked. In contrast, protocols in Rosetta3 are typically stand-alone applications with their own name. There are currently over 200 such applications.

Two scripting interfaces are also supported: *RosettaScripts*[18], which is an XML-based interface that allows protocol development from modular building blocks without additional compilation, and *PyRosetta*[19,20], which allows Python-based protocol development and easy integration with third-party tools. The different interfaces are rooted in the Rosetta3 codebase from 2007, which is still used, developed and maintained at the time of this writing, 12 years later. In our case, after several iterations we found appropriate reconfigurable and extensible abstractions within an object-oriented programming framework in the C++ language to achieve our scientific objectives.

### Version control

In software development, version control is important both to document changes to the software over time and to ensure that independent, parallel lines of development can be merged into a single, unified software. Version control is especially important for large codebases with complex dependencies of files and class hierarchies and where each revision in the main line of development needs to compile and run.

In the mid 1990s, our developers stored versions of the codebase in cryptically-named subdirectories. A single source code repository was created to which a single person had write permissions (Carol Rohl), using the then-popular Concurrent Versioning System (CVS[21]). CVS scaled poorly as the community of developers grew, and around 2006 the community moved to the centralized version control system Subversion (SVN[22]), which worked well for us for many years. As the number and geographic range of developers grew and the code complexity increased, our single SVN server became overwhelmed. Developers distant from the central server in Seattle encountered painfully slow or impossible merges due to timeouts, or had unsaved local changes erased. In 2013 we migrated to private repositories on GitHub[23], which uses the Git distributed version control system. Early on, we created conventions for interaction with GitHub (*e*.*g*. no forking, rewriting pushed commits, or force-pushing into the master branch). The decentralized nature of Git has served the community extremely well in supporting thousands of different development branches from all over the world that can be relatively easily merged into the main development branch. We have since instituted additional checkpoints for merges including automated testing (see below) and requiring independent code reviews from other developers in the community on each changeset (pull requests and reviews–see below). Pull request reviews have enabled us to improve code quality and reduce bugs. Administrative control of the code repository rests with senior developers, rather than principal investigators (PIs).

### Testing framework

**Fig 4:**
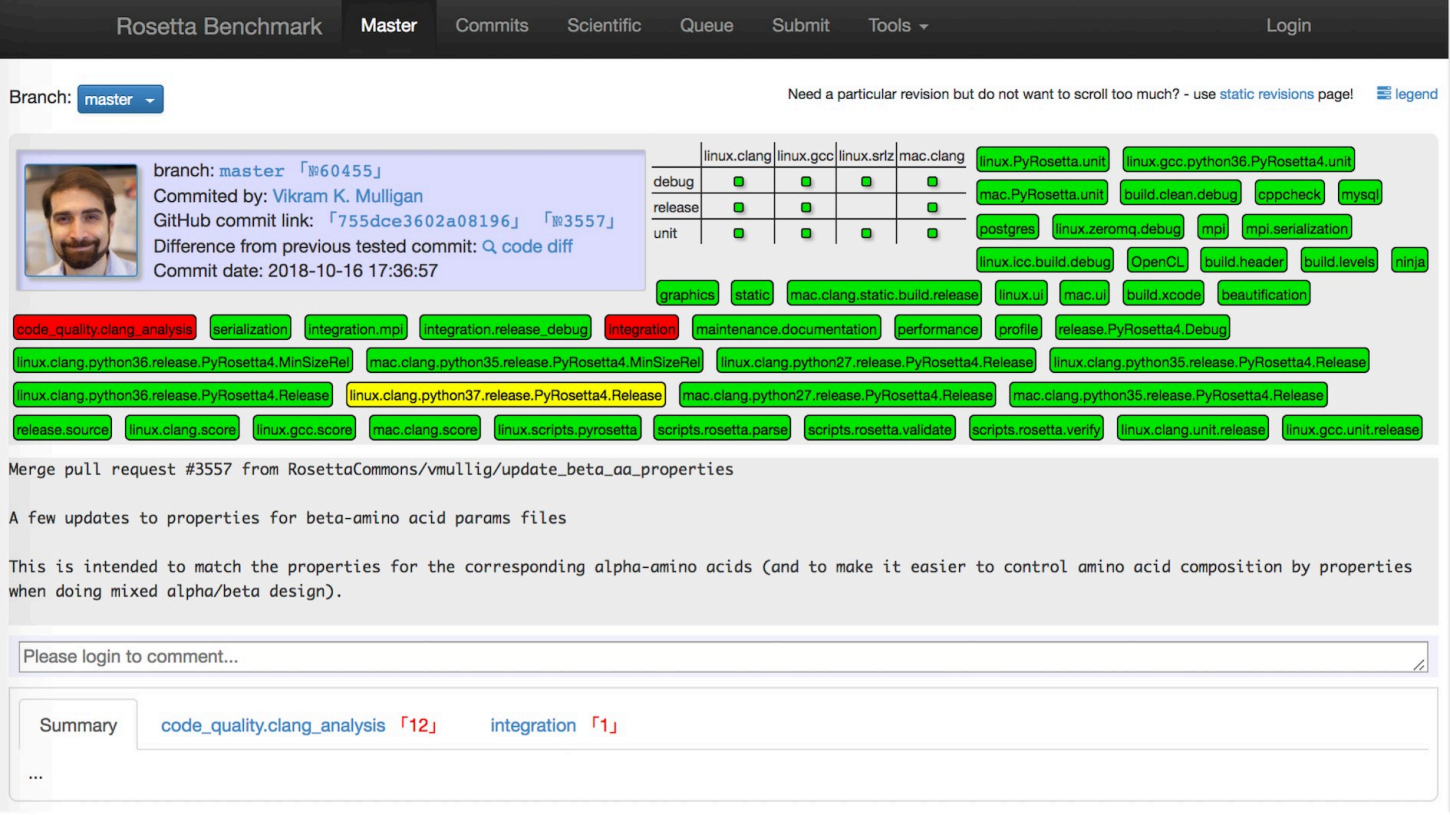
Software testing. Testing is essential to ensure stability and correctness of software. This is a view of our testing server dashboard for a specific merge into the master branch of Rosetta. We continuously run a large number of tests, such as build tests, unit tests, integration tests, performance tests, code analysis tests, and scientific benchmarks. Tags in green, red, and yellow denote test pass, failure, and currently running.

Code testing is necessary to ensure that software runs correctly and stably in different computing environments, provides expected and reproducible output, runs within a certain timeframe, and achieves some technical objective. Starting in 2004, we created our own testing framework because standard software packages at the time did not provide the features we needed. Testing has become an integral part of our software development. Because running tests locally on various architectures and build modes is challenging and time-consuming for individuals (see S1 Text), our framework includes a dedicated testing server on which tasks are distributed and results are collected (Fig 4). While establishing a custom test infrastructure might not be feasible for smaller software projects, several free and paid services are now available that are highly beneficial for scientific software (e.g. BuildBot[24], Jenkins[25], GitLab[26], TravisCI[27]). We support the following types of tests that are described in detail in the supplement: build tests, unit tests, integration (regression) tests, scientific tests, and others. RosettaCommons uses licensing revenue (see “Licensing” below) to acquire and maintain the hardware on which these tests are run.

### Improving code quality through code review

A requirement for collaboration and longevity of scientific software is a readable, maintainable, and extensible codebase as a foundation for new scientific development. In reality, however, everyone writes code differently. To create a base level of readability of the code, limit code breakage, and make code easier to debug and extend, we agreed on a set of about 200 coding conventions[28], to which everyone in the community is expected to adhere. Although it is challenging to strictly enforce all conventions, some are enforced by automated code quality tests, a custom “beautifier” script that formats code according to our conventions, and a Python-based code templating system for various common class types. Other conventions are socially enforced through code reviews (see below).

Several software development tools and frameworks have provided us with functionality that shaped our software development practices. We integrated features of Git and GitHub into our testing framework, including branching, pull requests, and code reviews (see S2 Text for workflow). A pull request is a request by a developer to merge new code from a branch into the main repository. The requestor can tag specific people to perform a review or broadcast a general call for reviewers. Based on the received comments, there can be multiple rounds of revisions. When all reviewers approve the merge, a final set of tests are run and the code is merged. This workflow is similar to software engineering industry practices and improves the quality of code contributions while also lowering the psychological barrier for new developers to contribute to the codebase without the worry of breaking it. In our experience, it can be challenging to maintain code quality, especially in large, diverse communities, and interactive code reviews have been a great benefit.

### Interfaces to the main codebase

To facilitate participation by diverse new contributors, methods can be written in C++[12], PyRosetta[19] or RosettaScripts[18]. The choice for the interface is often made by considering technical details of the new method as well as the experience and comfort level of the developer. Protocols in RosettaScripts or PyRosetta (for which the C++ implementation is the common foundation) can be developed more quickly and require less technical knowledge but are subject to API changes and are frequently not merged back into the main codebase.

The first, simplest, and most-used interface to the codebase is the command line. Other interfaces were implemented over the years for different purposes and to facilitate custom protocol development. For example, in 2008, the **Foldit** video game[29,30] was released, which uses Rosetta as a foundation and employs a graphical user interface to allow the player to manipulate a protein structure. Major scientific concepts were adapted with a simplified terminology to make Foldit accessible to a broader audience. For instance, restraints are termed “rubber bands”, “shake” is used for rotamer optimization and design, and “wiggle” for high-resolution refinement. The goal of the game is to achieve high scores rather than low energies. Foldit is widely used by the general public to solve real scientific puzzles[31], as a teaching tool for education levels from K-12 to graduate school[32], and by academic labs to gain insights and inspire new scientific discoveries.

In 2011 Fleishman *et al*. introduced **RosettaScripts**[18], which is an XML-based interface that allows custom protocol development by recombining and configuring existing objects. It takes advantage of the object-oriented framework of Rosetta3[12] and allows the user to create a new recipe without writing or compiling any C++ code. Intimate knowledge of the C++ codebase is not required, as the vast majority of the objects used in RosettaScripts are documented online[33]. Using RosettaScripts has been further facilitated by the XML Schema Definition (XSD) framework that identifies errors in the XML scripts at runtime, provides detailed error messages, and is now integrated into our user-facing documentation.

While RosettaScripts is useful for protocol development, it generally lacks flow control and detailed control of how objects can be manipulated. If these are needed and more specific control over certain objects are required for development, **PyRosetta** is the tool of choice. Since the early 2000’s, the Python language has become the dominant programming language in many scientific disciplines, including biology and chemistry; largely because it is easy to learn, forgoes code compilation and features automatic memory and package management, while performing better than many other scripting languages. After initial work by William Sheffler, in 2009, Jeffrey Gray’s group at Johns Hopkins University released PyRosetta[19,20], which enables the user to interact with nearly all underlying data structures *via* Python bindings (PyRosetta exposes 87,896 C++ functions and 7,403 C++ classes at the time of this writing). PyRosetta is for advanced users and beginning developers as it requires knowledge of the Rosetta libraries. A graduate course about protein structure modeling with PyRosetta is taught by Jeffrey Gray (videos are available at www.pyrosetta.org) and others in our community. Updated workshops are available on the website, and a book describing them was published in 2009[20].

Development in the C++ codebase is necessary for optimization of new protocols and the addition of new functionality and advanced features, such as new biomolecules or systems to model; examples include carbohydrates, nucleic acids, or symmetric systems.

Other interfaces include the CS-Rosetta toolbox[34,35] for incorporating NMR data from chemical shifts and residual dipolar couplings via Python wrapper tools, and InteractiveRosetta[36], which is a graphical user interface to PyRosetta[19].

## Social aspects

### The RosettaCommons community of developers–rewarding collaboration

The technical considerations outlined above reflect years of work and iteration by many scientific developers. As in many scientific software communities, the number of computer scientists and software engineers in our community is small compared to the number of scientists with domain expertise who have acquired programming skills without formal training. Developers go beyond applying existing code to current problems in that they also develop novel methods to address new scientific questions. By drawing from over 60 labs worldwide, the community includes developers with backgrounds in biochemistry, chemistry, physics, biology, computer science, mathematics, engineering, pharmacology, molecular physiology and other, related disciplines at the undergraduate, graduate, postdoctoral, and career professional levels (research scientists, principal investigators), and their collaborators. To fill these gaps in expertise (inevitable due to the large scope of the code) and work effectively together, we foster a culture that encourages communication, training, leadership, and outreach.

### Achieving a robust codebase with scientific outcomes requires collaboration

Computer scientists and software engineers (especially in commercial settings) often emphasize correctness, code design, maintenance, readability, and performance. In contrast, academic scientists often favor rapid development of “messy” code to test a hypothesis, and scientists without formal computer science training emphasize scientific correctness and learn about software engineering concepts later or never. These factors lead to a codebase that is difficult to read, extend, maintain, and test, and which often performs poorly. In our community, we actively encourage, inspire, and teach these concepts to build a culture that appreciates and actively participates in code maintenance. To combat these barriers, sustainable software development requires (1) periodic code maintenance and (2) a culture of appreciation for software engineering concepts. We reinforce such a culture by giving out an annual *Rosetta Service Award*, which over the past ten years has honored contributions from code refactoring, hackathon leadership, diversity and inclusion efforts, and more. Anchoring these values into our community enables us to save a lot of time, effort, and money while interacting closely with community members allows us to implement new ideas more rapidly.

### RosettaCommons membership

A central question is: who is the RosettaCommons? We seek to include all who share our scientific goals to develop Rosetta to predict structures and design macromolecules. In practice, the “community” intellectual property (IP), that is the code itself but not the products of the software, requires a legal sign-on from individual developers and their host institutions. The institutions and developers agree to contribute their intellectual property to the RosettaCommons codebase, to provide for an environment of continuous sharing and collaborative development for the advancement of public science. PIs are invited leaders of research groups, and developers in those groups sign a developer agreement. Historically, new PIs have been alumni from David Baker’s and other first-generation RosettaCommons labs. As our platform has been used in other areas of science (e.g. cryo-EM reconstruction or nanomaterials), new members have joined without training in specific Rosetta groups. The RosettaCommons executive board is currently evaluating possible strategies on how to expand the RosettaCommons to properly include the expanding set of developers.

### Leadership: RosettaCommons Executive Board

The RosettaCommons was established in 2001[8] to facilitate collaboration between our laboratories at various institutions, and manage intellectual property and licensing of the codebase in collaboration with the University of Washington’s technology transfer office, called UW CoMotion. Executive decisions are made by the principal investigators either at the *PI meeting* at the annual *RosettaCon* or via an email list. Since the RosettaCommons has grown substantially, we created an **executive board** in 2014 to oversee the community service work from communications to documentation, awards and budgets. PIs are elected to two-year terms for various board positions to match their expertise or interests, except for the conference chairs which rotate annually. The descriptions of the board positions can be found in S3 Text.

### Communication

As our software is developed by many labs worldwide, constant and efficient communication is crucial. Several **mailing lists** are in place for the C++ and PyRosetta developers, the PIs, test server logs to receive updates on code breakage, the diversity committee, and a jobs list, among others. We also have a **Slack** workspace for more interactive communication and for fast support within our community. We further have a **MediaWiki**[37] since the early 2000’s communicating announcements for conferences and teleconferences. Most of the documentation has moved to a **Gollum wiki**[38], discussed under documentation below. Users can also ask questions on a searchable forum[39]. Monthly ***TeleCons*** allow the developers, the PIs, and the diversity committee to communicate. The variety of communication channels serve different purposes and further create a sense of community.

### Community interaction in meetings and hackathons

The RosettaCommons spirit of close collaboration is fostered by regular meetings, the most significant of which is the annual ***RosettaCon*** in the summer. The inaugural meeting in 2003 was similar to a lab retreat, spending a few days in the North Cascades to share new developments. The last day of the meeting has traditionally been reserved for leisure activities as an opportunity to get to know the members of the community. Talking about science in a more relaxed setting has been a boon to our community spirit. For many people, ***RosettaCon*** feels more like friends solving scientific and technical problems together rather than a traditional scientific conference.

**Fig 5:**
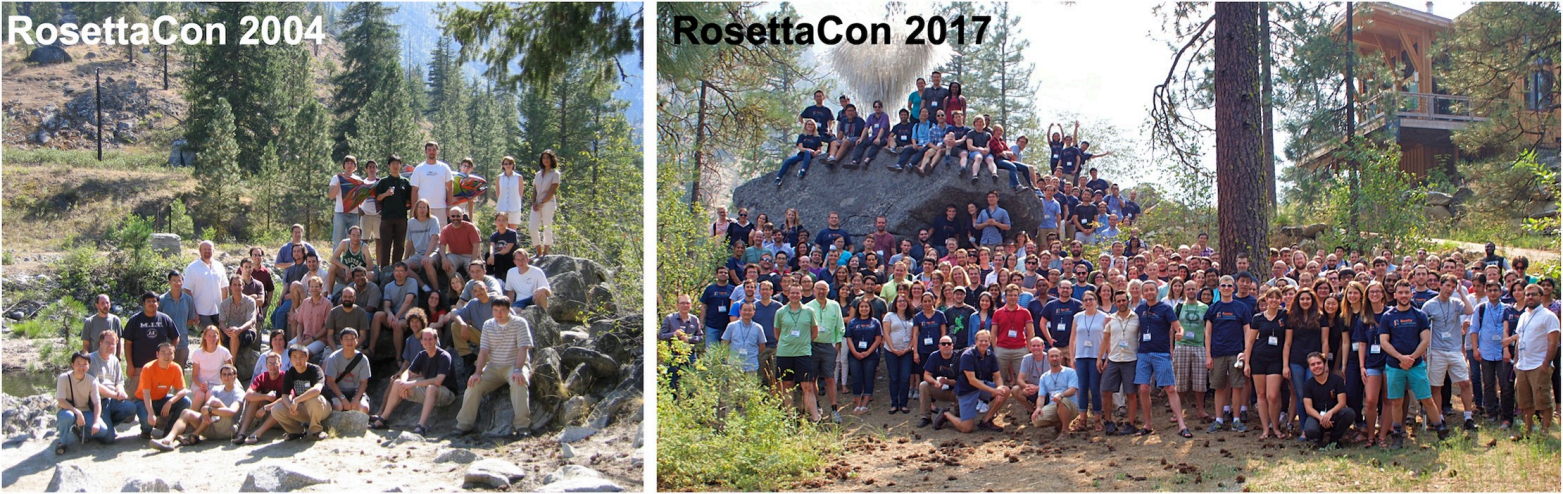
Meetings. Developers meet at our annual summer conference (RosettaCon). Pictures from our second RosettaCon in 2004 (left) and the conference in 2017 (right) reflect the community’s drastic growth and increase in diversity.

Summer ***RosettaCon*** has grown significantly to 270 people by 2017 (Fig 5). As the audience grew, the conference has shifted from mostly software discussion to also include scientific developments. All attendees present their research in a poster session or talk. The quality of the talks has increased substantially from less-formal talks (similar to group meetings) to well-polished presentations one would expect at international scientific conferences. Speakers range across all career levels.

In-depth code developments are discussed at ***PreRosettaCon*** (formerly called the *developer’s meeting*) where major decisions about code maintenance, improvements, and restructuring are made. These discussions can be very technical and organizational. Senior developers typically organize the meeting, with decisions being made democratically by all attendees. We further organize an additional meeting, ***WinterRosettaCon***, in February (formerly called *developer's meeting* or *MiniCon*, which started in 2007) with rotating locations. Similar to *PreRosettaCon*, these meetings have been smaller and more technical, covering both technical talks as well as discussion time for major decisions. In the last few years, however, *WinterRosettaCon* has grown to include a *bona fide* scientific program.

**Fig 6:**
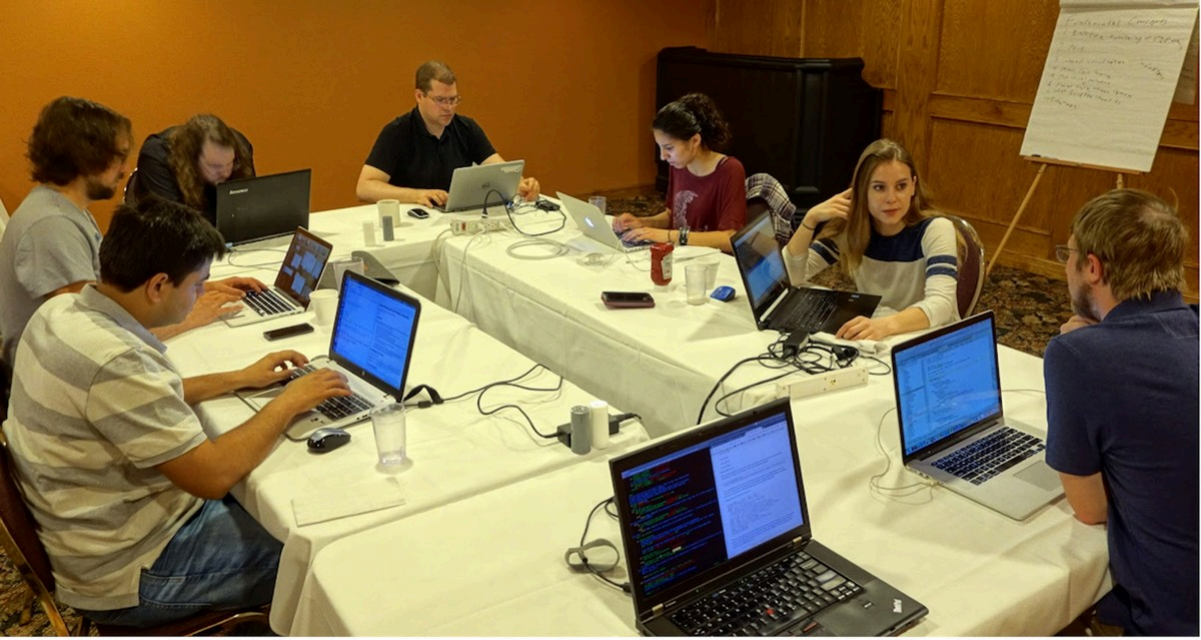
Hackathons. Organizing regular hackathons can drastically improve code maintainability, generalizability, documentation, and interaction in the community. Our eXtreme Rosetta Workshops (XRWs) are organized annually and have had a drastic positive impact on our software and community.

The community further organizes on-demand hackathons (***XRWs***–*e****X****treme* ***R****osetta* ***W****orkshops*) for maintenance and improvement of the codebase. In these *XRWs*, about ten people from different research groups meet at one location to accomplish a specific, well-defined goal. Previous *XRWs* focused on improving compilation time and memory use by splitting the core libraries (2010) and the protocol libraries (2011); improving the scorefunction (Talaris meeting 2013 –this was technically not an XRW hackathon but a one-off technical meeting with presentations and discussions); improving documentation, protocol captures, and tutorials (2015 and 2016 –Fig 6); generalizing storage of chemical information and handling of common molecular file formats (2016); improving the handling of RosettaScripts input (2016); and generalizing interfaces for handling non-canonical amino acids, carbohydrates, nucleic acids, and metal-binding sites (2017). *XRWs* have had a tremendous impact on maintainability, extensibility, and user-friendliness of our codebase while further enhancing community spirit between participants.

### Training for users and developers

Rosetta is written with the goal of understanding complicated molecular systems at the interface between chemistry, physics, and biology. Since the software has primarily been developed by scientists rather than software engineers, it has historically been difficult to use. The creation of various user and developer interfaces (command line, PyRosetta, RosettaScripts) is a result of trying to address some of these challenges. In addition, our community has established a number of workshops to make novices familiar with the use of the software suite and its codebase as quickly as possible.

Users with a general understanding of protein science seeking an introduction to commonly-used protocols can follow a set of **tutorials** compiled by the lab of Jens Meiler and collaborating developers at Vanderbilt University; they supplement a manuscript describing the main protocols[9]. These tutorials are taught twice a year at Vanderbilt[40], and are probably the quickest way to learn how to use the software.

In 2013, we first began offering a week-long ***Bootcamp*** course (now called Code School) for small groups of interested graduate students and postdocs in RosettaCommons laboratories. The course covers intricacies of C++ programming and the architecture of our software libraries in lectures and in-class labs. We keep a high ratio of teachers to students (~1:5) so that multiple teachers can answer questions simultaneously during the lab sessions. The course was intended to train non-computer scientists to become senior developers and hopefully address our gender imbalance (more below). Videos of code school lessons are available on the RosettaCommons YouTube channel[41]. In 2019, we added a ***PyRosetta Code School*** course, which has an even lower barrier to entry as Python is an easier language to work with. *Code Schools* directly taught by senior developers have drastically improved code quality, consistency, readability, documentation, and understanding of important software engineering concepts. They have further improved the interaction and communication within the community and helped newcomers feel accepted.

Because gaining proficiency in software development typically takes many months to years of training, some members move between RosettaCommons laboratories throughout their career to apply their skills to different types of macromolecular modeling and design problems. These individuals, often having 6+ years of experience and institutional memory, play a crucial role in advancing and supporting the codebase, our community and training newcomers. Further, the RosettaCommons supports two research professors (Andrew Leaver-Fay and Rocco Moretti) who use their expertise to answer difficult technical questions and who are qualified to make major decisions about the codebase, and an experienced senior test engineer (Sergey Lyskov) who created and maintains the test infrastructure. Collectively, the 15–20 senior developers, scattered across multiple labs, have comprehensive knowledge of the ever-growing codebase and functionality of the software suite.

### Diversity, Inclusion, and Outreach

Building and maintaining a strong community of collaborators requires fostering and recruiting top talent. As our community grew, we noticed that our team lacked gender and racial/ethnic diversity and that we were missing a sizeable fraction of the talent available. We believe an inclusive culture and a diverse membership are prerequisites to forming an ethical and just community of scientists, so we focused on recruiting and developing the scientists we failed to include previously.

In addition to offering *Code Schools*, two important first steps were instituting a Code of Conduct[42] and forging a diversity statement[43] to capture our values and aspirations. The Code of Conduct includes a reporting system that allows us to refer legal issues to home institutions and to address microaggressions at conferences on-site. The diversity statement[43] commits to making “our community inclusive and equitable for people of all backgrounds, regardless of race, ethnicity, nationality, gender identity, sexual orientation, disability status, age, belief system, or socioeconomic background” (see S4 Text). It also “urges the individual research groups in our community to make every effort to proactively and swiftly eliminate discriminatory policies and procedures in hiring and recruitment and to make efforts to increase the number of individuals from historically underrepresented groups in the Rosetta community”.

As specific actionable items, RosettaCommons members have participated in a variety of outreach activities that fulfill several roles: (1) educating the general public about science, (2) sparking interest in STEM in K-12 and undergraduate students, (3) increasing diversity and inclusivity in the RosettaCommons, and (4) crowdsourcing scientific discoveries.

Our recruitment efforts include regular attendance at conferences that aim to highlight diversity in STEM fields such as the *Grace Hopper Celebration of Women in Computing*, the *Annual Biomedical Research Conference for Minority Students* (ABRCMS), the *ACM Richard Tapia Celebration of Diversity in Computing*, and the *Society for Advancement of Chicanos/Hispanics and Native Americans in Science (SACNAS)* conference. These conferences directly strengthen our climate and culture, as solo-status[44] Rosetta scientists in their home lab meet in person with those sharing their identities in other RosettaCommons labs and beyond, forging ties and networks that endure past these meetings. Professional development opportunities at these conferences empower scientists from minority groups and enlighten delegates from majority groups.

For undergraduates interested in a research experience in a RosettaCommons lab, we have established an NSF-funded REU (Research Experience for Undergraduates) program, the *Rosetta Summer Intern Program*[45]. Our program starts with a week-long *Intern Code School* where students learn how to use the software, how to navigate the code, and the most important frameworks[46]. After that week, students are able to develop basic protocols and accomplish specific research goals during their 8-week internship, results of which they present at RosettaCon.

Additional activities to improve inclusion in our community have included: (1) creating an active Diversity and Inclusion Committee (see Rosetta consortium supplement for members); (2) data collection on conference demographics; (3) invited platform speakers at RosettaCon to discuss diversity topics (Karen Fleming on gender equity, John Matsui on diversity 101, Joyce Yen on leadership and measuring inclusion); (4) networking/mentoring events at RosettaCon for women and members of minority groups; (5) RosettaCommons climate survey and follow-up small group discussions at RosettaCon resulting in recommendations and new actionable items; (6) dependent-care grants to support parents to attend Rosetta meetings; (7) conference housing matchups considering non-binary gender; (8) collecting resources for career development and personal well-being.

Our efforts have substantially increased the number of women in a typically male dominated field. Nearly 30% of *RosettaCon* attendees in 2017 identified as female, up from below 15% in most years before 2012 (S1 Fig). Almost 8% of attendees identify as Black, Hispanic, or multiracial. The demographics of our core developers and PIs are much less diverse, giving us humility and recognition that diversity efforts need to be sustained for many years to come. We will continue our work to ensure the RosettaCommons is a universally welcoming and supportive environment.

### Public participation in science

In the early 2000s when protein structure prediction was much less tractable and compute resources were limited, we created a distributed computing project called **Rosetta@home**, through which volunteers can donate idle computer cycles for protein structure prediction, docking, and design[47,48]. Rosetta@home was initially released in 2005 and runs on the Berkeley Open Infrastructure for Network Computing (BOINC)[49]. Rosetta@home shows a screensaver while performing computations when the user’s desktop or laptop is otherwise idle. In 2015, Rosetta@home was ported to the Android operating system to run on volunteers’ cell phones and mobile devices during recharge periods, when the CPU is typically underutilized[50], to perform biomolecular structure prediction tasks.

Similarly, the **Human Proteome Folding Project**[51] was a combined effort between two Rosetta labs, the *Institute for Systems Biology*, and IBM’s *World Community Grid*. It was launched in 2004 with the goal to predict protein structures in the human genome, running for 9 years. Its successor, the *Microbiome Immunity Project*[52] was launched in 2017 with the goal to predict protein structures from the human gut microbiome.

## Dissemination and interaction with users

### Packaging and platforms

Rosetta simulations typically require High Performance Computing (HPC) resources, which are often public or university-owned. In response to the heterogeneity of available HPC resources, our software must be buildable across a wide range of hardware and compute environments. We customized the build system SCons[53] to allow users to specify system-specific compilation settings and select a particular build mode (e.g. a *Message Passing Interface* (MPI) mode for clusters that support or require it). Precompiled binary builds for 64-bit Linux and Mac distributions are available along with weekly source code releases (since 2013). Larger, numbered releases occur once or twice annually. The software suite runs on the three major operating systems (Linux, macOS, and Windows through the Windows Subsystem for Linux) and hardware architectures currently in use (x86-64 Intel/AMD chips, IBM Power architecture [e.g. Blue Gene supercomputers], and ARM for android-based cell phones and tablets [for Rosetta@home]). In the past, DEC Alpha, Cray, and Intel Itanium architectures were also supported.

### Webservers

One path to perform simulations as described in published manuscripts is through webservers that present a limited number of parameters to the user. The Robetta server[54] was deployed in 2003 and is commonly used by scientists inside and outside of our community for fragment generation[55], domain parsing and structure prediction[56,57], alanine scanning[58], and DNA interface scanning[59]. Individual labs have also created servers for design[60], flexible backbone modeling and design[61], flexible peptide docking[62], and protein-protein interface identification[63]. In 2013, the ROSIE server framework[64] was created to simplify setting up new webservers for Rosetta applications. As of 2019, ROSIE hosts 21 modeling protocols, has >7,300 registered users and has run >75,000 jobs for a total of nearly 7.8 million CPU hours.

### Licensing and commercialization

When developing software, it is important to think about licensing to answer the following questions: (1) by whom and under what circumstances will the software be used?; (2) how will contributions to the codebase be made, and from whom will they be accepted?; (3) will revenue be generated and, if so, how and what will it be spent on?; and, (4) will the choice of license restrict the use of external software libraries?

The Rosetta software is released under a unique licensing model, tied to its history[8]. Initially, when the software was developed solely by students and postdocs in David Baker’s lab at the University of Washington, it was clear that sharing openly and avoiding disputes over ownership enabled innovative, rapid scientific progress. As these trainees started their own research groups at other institutions, they sought a licensing model to preserve the collaborative spirit and encourage sharing by removing barriers to inter-institutional development, leading to the establishment of the RosettaCommons. By joining the RosettaCommons, member institutions agree to provide non-exclusive rights to the copyright for contributions made to the codebase, and developers sign an agreement assigning copyright of code written by them to their institution. The software is distributed free of charge to academic, governmental, and other non-profit institutions. Commercial entities support continued development by paying a reasonable licensing fee that is based on the number of employees (more detail in S5 Text).

Revenue from commercial licenses, as well as government or private funding, is used to support meetings, software maintenance, outreach activities, trainings, lab exchanges and hackathons, mini-grants, and service awards to incentivize and reward community support. RosettaCommons PIs can apply for mini-grants, which are typically awarded to maintain, improve, extend and support the codebase, for instance for energy function improvements, rewrites of major frameworks, development of user interfaces and documentation, etc.

Member institutions of the RosettaCommons collectively hold the copyright to the source code but make no claim to the IP associated with products derived via use of the software (*e*.*g*. new protein therapeutics). Such IP is owned by the licensee, whether such licensee is academic or commercial. This has allowed spin-off companies to flourish that either use our software themselves under a commercial license to perform research and develop products/services, or such companies have licensed IP created by Rosetta academic licensees to allow product development. There are currently several commercial entities based entirely or in-part on our software, including the Rosetta Design Group, Arzeda, Cyrus Biotechnology, PvP Biologics, Neoleukin Therapeutics, and Lyell Immunopharma (see S6 Text).

### Documentation

Rosetta is an inherently complex piece of software written for and by experts in the field, which can result in a complicated interface with lots of options. To ensure that scientists can use the software effectively, our developers have emphasized robust documentation with example workflows of real-world use cases called protocol captures[8,9]. Protocol captures force a developer to consider the perspective of end-users who may have different biological problems to solve, a differing scientific mindset, and different technical skills. Since scientifically focused grants (for instance from government agencies) typically do not provide time for code maintenance and writing documentation, the mini-grants, XRWs, and training or service awards help incentivize and recognize this important work.

Over the years, our community has used several types of documentation. To assist developers, the coding conventions for C++ (and later Python) include the use of comment-based API-level in-code documentation (**Doxygen**[65]). The combination of coding conventions with the requirement to write in-code documentation facilitates reading and understanding code written by someone else. We recently developed a Python-based code template system for frequently used classes to continue to improve both consistency and code commenting, while significantly reducing development and debugging time. While in-code Doxygen documentation is useful for developers, it is less beneficial to end-users because it describes internal functions and not the interface to an executable or RosettaScripts component.

Our user base spans thousands of users who require support, and our developers become users once they run someone else’s code. This provides an incentive to write effective documentation[38], tutorials and protocol captures to answer common questions. Our documentation was transferred from an initial Doxygen-based[65] collection of application documentation pages to a **Gollum wiki**[38] and then greatly expanded upon during two *XRWs*. The Gollum wiki is split into user-facing and developer-facing documentation. The former contains information about released code, tutorials and generally helpful information for our users. The latter documentation contains information about experimental features pre-release and technical information. Both user-facing and developer-facing documentation are easily editable by RosettaCommons members and up-to-date with the current version of the software; as such, its detail, accuracy, and searchability have been transformative for both users and developers. This effort, combined with a push in the community for **protocol captures** in the supplementary material of published papers, have drastically improved quantity and quality of documentation. Another starting point for users is reviewing command lines used in the provided tests or demos for various applications. In addition to documentation, we have focused on improving error messages, for instance *via* XSD in RosettaScripts, to help users debug their custom scripts. Not all questions and use cases can be anticipated, so we also have a forum[39] to provide support and use the feedback from our collaborators to advance the software.

## Remaining challenges

Although we have addressed many problems over the last 20 years, there are still several outstanding issues with respect to the three main areas explored in this paper: (1) Technical: The tension between best practices in software development and rapid scientific progress means we must continually provide incentives for prioritizing maintenance, usability, and reproducibility. Additionally, we are currently reconsidering the basic abstractions and data structures that we have relied on for over ten years to make use of massively parallel hardware architectures (e.g. GPUs). (2) Social: The enormous growth of our community contributes to questions of community and lab membership, the effectiveness of our diversity, equity, and inclusivity efforts, how to preserve the small, informal vibe as we continue to grow, and how to appropriately give credit (i.e. authorship) in an environment that is so clearly a team effort. (3) Dissemination: The diversity of applications, workflows and computer architectures make it challenging to ensure that outside users can easily integrate Rosetta into their workflows, and provide resources for backend compute for web servers, targeting a broader range of scientists. Fifteen specific challenges are detailed in S7 Text.

## Conclusions

Here we use the Rosetta software as an example to highlight the successes, mistakes made, and lessons learned over the past two decades as we developed a scientific software tool in a global, diverse, and distributed community. Our software was created mostly in an academic environment and is widely used beyond the laboratories in which it was created. We demonstrate the necessity of mastering the technical aspects of software development to create a package that allows the use of complex tools to drive the cutting edge in computational structural biology research.

Reasons for success in our community are numerous and largely intertwined. First and foremost, our community has lowered the barriers for scientific collaboration and thus enabled groundbreaking science and faster implementation of good ideas. On the technical side, we benefited from early established coding conventions, version control on GitHub, and extensive code-testing through an integrated framework. We further maintain close interactions with users inside and outside of our community, who enable us to think outside of our established patterns. Understanding user needs and how our tools are actually used (instead of how we think that they are used) drives us to continuously improve our software for application to “real-world” scientific questions. To best take advantage of our broad developer community and to best support the users in our community, we created easily editable documentation that proved transformative in completing the coverage of the documentation and in supporting our large external user base.

Most importantly, scientific software cannot be sustained without a community around it, and we rely heavily on our community for training, communication, hackathons, and sharing expertise, whether scientific or related to software engineering. With the RosettaCommons, we have established an open community of developers that thrives on collaboration instead of competition: our common goal is to advance science. We are not afraid of tackling the most challenging scientific questions; in fact, we are motivated by them. Because our community is broad with a flat hierarchy, members have a voice irrespective of career level and are encouraged to contribute. Because of this, our community has established its own agreed-upon standards that members abide by. Forming such a tight-knit and collaborative community would not have been possible without a solid licensing model that was the result of early, thoughtful decisions that benefitted both our software and community over time. The agreement signed by all of the institutional members and developers of the RosettaCommons enables us to share and develop code independent of institution and commercial licensing of Rosetta allows industry to indirectly support maintenance and continued development through licensing fees.

Science is increasingly performed in teams to address complex questions with advanced methods and there is a need to address the challenges of achieving scientific goals in large groups[1]. The challenges we faced are remarkably similar to those noted by the National Academy’s Committee on Team Science[1]; we believe that these actions and lessons are independent of scientific discipline and therefore transferable to other communities. Our diverse community has thrived by committing to our shared goals and strong belief that, in fact, we are better together.

## Supporting information

S1 Fig Growth in Rosetta PIs and conference attendees. (DOCX)

S1 Text Specific tests for Rosetta running on our automated testing framework. (DOCX)

S2 Text Typical development workflow in the Rosetta community. (DOCX)

S3 Text Descriptions of the Rosetta board positions. (DOCX)

S4 Text Values Statement: Equality and Inclusion in the RosettaCommons. (DOCX)

S5 Text More detail on specific licenses. (DOCX)

S6 Text Commercial spin-off companies from the Rosetta community. (DOCX)

S7 Text Details of Remaining Collective Challenges. (DOCX)

S1 List Members of the RosettaCommons Consortium. (DOCX)
