# Supplementary material for: Better together: Elements of successful scientific software development in a distributed collaborative community
Source: PLoS Comput Biol. 2020 May 4;16(5):e1007507. doi: 10.1371/journal.pcbi.1007507 (PMC7197760; doi:10.1371/journal.pcbi.1007507)

***S1 Fig: Growth in (a) Rosetta PIs and (b) Conference attendees.***

2013 conference data were unavailable. In 2017, additional housing was secured off-site to increase the meeting capacity. 2018 RosettaCon counts include one person who identifies as non-binary gender.

1.
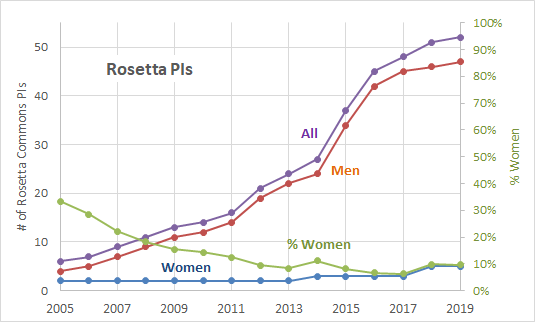

Supplement: S1 Fig — (DOCX) [file pcbi.1007507.s001.docx]
