# Supplementary material for: Better together: Elements of successful scientific software development in a distributed collaborative community
Source: PLoS Comput Biol. 2020 May 4;16(5):e1007507. doi: 10.1371/journal.pcbi.1007507 (PMC7197760; doi:10.1371/journal.pcbi.1007507)
Supplement: S1 Text — (DOCX) [file pcbi.1007507.s002.docx]

1.
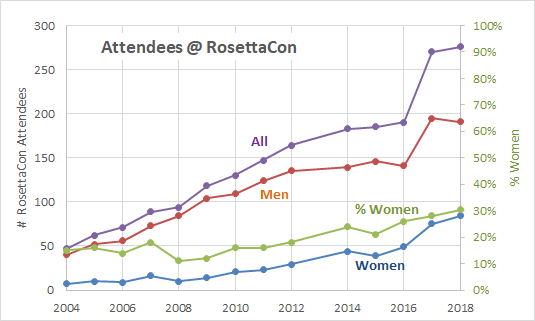


## S1 Text: Specific tests for Rosetta running on our automated testing framework:

(1) **Build tests** ensure the software can be built in various operating systems (Linux, macOS, Windows (through the Windows Subsystem for Linux), modes (release, debug, MPI, statically-linked, serialization, and modes for output into MySQL or Postgres databases), hardware architectures, and compilers/build tools (GCC, Clang, ninja+cmake, SCons). Our build tests have run as pass/fail tests since 2007.

(2) **Unit tests** are pass/fail tests that ensure that individual “units” of code work correctly, *i.e.* give the correct output given some input variables. For example, a sum function given two input variables with values 3 and 5 should return the value 8. Unit tests should be written in a way to test for all envisioned edge cases. We have run unit tests using the CxxTest suite[1] since 2007.

(3) **Integration tests** make sure that different code objects and functions can be integrated into a single application. For example, one would test the protein design application on one specific set of inputs, creating a single protein model. Our integration tests are run as regression tests, meaning that they don’t test for correctness of the output, instead identifying changes compared to previous versions. Our criteria include a maximum execution time of 30 seconds and completion without error, given a set of reasonable inputs. We started running continuous integration tests on our first test server in 2004.

(4) **Scientific tests** ensure that individual applications meet their scientific objective. If we claim that *de novo* structure prediction produces a meaningful model in 30% of the target cases, we need to test a benchmark set and create about 10,000 models for each test case to verify that this claim holds true. These tests require substantial amount of compute time. In our community, scientific tests (also called benchmarks) are typically performed individually as part of a publication describing a new or improved application[2]. They represent the outcome at a specific time and are not run periodically. The disadvantage of this approach is that we are unaware if or how daily changes to the codebase (from ~100 active developers) affect the scientific validity of established protocols. We are currently reintroducing continuous scientific tests on our test server. A previous attempt atrophied for various reasons: these tests require scientific expertise to set up, are difficult to write, hard to interpret with a pass/failure for a non-expert and since they are run infrequently, tend to get ignored. Having learned from these challenges, we hope to ensure longevity of these tests by running them on the centralized test server, nominating dedicated observers and keeping them up-to-date, more distributed responsibilities in updating these tests, improved documentation, organization and maintenance.

(5) Other tests we apply include code quality and coding convention tests (clang static analysis, “beautification”) and profile/performance tests (memory usage and runtime, for instance using Valgrind[3]).
