# Supplementary material for: Better together: Elements of successful scientific software development in a distributed collaborative community
Source: PLoS Comput Biol. 2020 May 4;16(5):e1007507. doi: 10.1371/journal.pcbi.1007507 (PMC7197760; doi:10.1371/journal.pcbi.1007507)
Supplement: S2 Text — (DOCX) [file pcbi.1007507.s003.docx]

## S2 Text: Typical development workflow in the Rosetta community

A typical development workflow now involves the following steps:

1. create a local and remotely tracked feature branch,
2. implement the feature in that branch,
3. create a pull request through the GitHub web interface,
4. schedule tests for the feature branch on our server,
5. request an independent code review,
6. at least one other developer reviews the code and requests changes (sometimes including new tests) as needed,
7. the original developer(s) addresses these requests (which may require help from the community), and commits the changes to the feature branch – the pull request is automatically updated,
8. once the tests pass and the reviewer(s) approve(s), the feature branch can be merged into the master branch.
9. The changes are automatically tested once more, in the context of the master branch, after merging. This ensures that small changes to the master branch that may have occurred since the feature branch was tested do not conflict with the new feature.
