# Supplementary material for: Better together: Elements of successful scientific software development in a distributed collaborative community
Source: PLoS Comput Biol. 2020 May 4;16(5):e1007507. doi: 10.1371/journal.pcbi.1007507 (PMC7197760; doi:10.1371/journal.pcbi.1007507)
Supplement: S3 Text — (DOCX) [file pcbi.1007507.s004.docx]

## S3 Text: Descriptions of the Rosetta board positions

Besides the Director (David Baker), other board positions are:

- Secretary – responsible for fostering communication and record keeping;
- Treasurer – responsible for managing licensing revenue and finances;
- Web and Social Media Chair – responsible for webpage and social media presence;
- Documentation Chair – to establish and update comprehensive documentation;
- Membership Chair – manages acceptance of new RosettaCommons labs and member institutions, maintains member list and policies;
- Awards Chair – manages *RosettaCon* awards and Rosetta Service Awards;
- Business Development Chair – manages interaction between RosettaCommons and commercial licensees and policies;
- Diversity Chair – manages the diversity and inclusivity efforts in the RosettaCommons;
- Conference Chairs – two PIs annually that organize the annual *RosettaCon*;
- At-large member(s) – advocate the interests of people not on the board;
- Grants Chair – manages distribution of Rosetta Mini Grants (typically ~$50k) awarded by the RosettaCommons.
