# Supplementary material for: Better together: Elements of successful scientific software development in a distributed collaborative community
Source: PLoS Comput Biol. 2020 May 4;16(5):e1007507. doi: 10.1371/journal.pcbi.1007507 (PMC7197760; doi:10.1371/journal.pcbi.1007507)
Supplement: S4 Text — (DOCX) [file pcbi.1007507.s005.docx]

## S4 Text: Values Statement: Equality and Inclusion in the RosettaCommons

The following statement was composed in a collaborative process, led by the RosettaCommons Diversity Committee and ratified by vote of all Rosetta PIs in 2018. The statement, as shared on the RosettaCommons website, is:

The RosettaCommons exists to enable shared developments and discoveries. We believe in the power of community to accelerate science. We strive to make our community inclusive and equitable for people of all backgrounds, regardless of race, ethnicity, nationality, gender identity, sexual orientation, disability status, age, belief system, and socioeconomic background.

The RosettaCommons urges the individual research groups in our community to make every effort to proactively and swiftly eliminate discriminatory policies and procedures in hiring and recruitment and to make efforts to increase the number of individuals from historically underrepresented groups in the Rosetta community. It should be a high priority for everyone in the RosettaCommons and the individual research groups to provide a welcoming workplace environment that is physically accessible to all and free of harassment and bias, both implicit and explicit.

These hiring and workplace practices are undertaken first and foremost because they are required to maintain an ethical and just community of scientists. Further, a diverse community increases the likelihood of novel approaches and applications of Rosetta science and enriches the experience of all members. Lastly, measures to increase the diversity and fairness of our research community will ensure that the next generation of scientists is drawn from all groups of people, resulting in a robust workforce on which the future of science and our society depend.
