# Supplementary material for: Better together: Elements of successful scientific software development in a distributed collaborative community
Source: PLoS Comput Biol. 2020 May 4;16(5):e1007507. doi: 10.1371/journal.pcbi.1007507 (PMC7197760; doi:10.1371/journal.pcbi.1007507)
Supplement: S5 Text — (DOCX) [file pcbi.1007507.s006.docx]

## S5 Text: More detail on specific licenses

Our licensing terms do not allow redistribution of modified or unmodified versions of the code but do not restrict or prevent licensees from modifying their copy of the source code. Since redistribution is prohibited, external additions to the Rosetta code libraries are generally incompatible with some popular licenses that enforce redistribution, such as GPL (GNU Public License) or licenses that use “share-alike” (SA) clauses. As Rosetta is commercially licensed, external additions under Creative Commons licenses are incompatible if they contain non-commercial (NC) clauses. Other commonly-used licenses (such as Apache and BSD licenses) may or may not be compatible with Rosetta as they depend on the specific restrictions and requirements of that particular license and require approval from UW CoMotion.
