# Supplementary material for: Better together: Elements of successful scientific software development in a distributed collaborative community
Source: PLoS Comput Biol. 2020 May 4;16(5):e1007507. doi: 10.1371/journal.pcbi.1007507 (PMC7197760; doi:10.1371/journal.pcbi.1007507)
Supplement: S6 Text — (DOCX) [file pcbi.1007507.s007.docx]

## S6 Text: Commercial spin-off companies from the Rosetta community

- Prospect Genomics (San Francisco, CA, 1999 - acquired by Eli Lilly / Structural GenomiX in 2001) Focused on genomics and drug discovery.
- Bio Architecture Labs (2007 - 2014) - Biofuels and biomass utilization using a combination of synthetic biology and enzyme engineering.

<https://xconomy.com/seattle/2009/04/02/uws-protein-guru-david-baker-eyes-alternative-biofuels-vaccines-in-new-3-d-structures/>

- Arzeda ([www.arzeda.com](http://www.arzeda.com), Seattle, WA, 2009 - current) offers enzyme design and metabolic engineering services.
- Rosetta Design Group ([www.rosettadesigngroup.com](http://www.rosettadesigngroup.com), Burlington, VT, 2009 - current) performs contract research and development, on-site client Rosetta-based research infrastructure development and training, and co-hosts RosettaCon with the University of Washington.
- PvP Biologics ([www.pvpbio.com](http://www.pvpbio.com), Seattle, WA, 2013 - current) is developing the Rosetta-designed enzyme KumaMax that breaks down gluten in the stomach to treat celiac disease[4,5], which is currently undergoing clinical trials.
- CompuVax ([www.compuvax.com](http://www.compuvax.com); Del Mar, CA, 2013 - current) uses computational antigen and antibody engineering to design vaccines for respiratory viruses.
- Cyrus Biotechnology ([www.cyrusbio.com](http://www.cyrusbio.com), Seattle, WA, 2014 - current) offers a web-based user-friendly interface of specific applications (under a specific license that Cyrus has negotiated with UW CoMotion in consultation with the RosettaCommons).
- Icosavax (Seattle, WA, 2017 - current) develops nanoparticle vaccines for infectious diseases.
- A-Alpha Bio (<https://www.aalphabio.com/>; 2017 - current) efficiently screens multi-specificity for drug development.
- Neoleukin Therapeutics ([www.neoleukin.com](http://www.neoleukin.com), Seattle, WA, 2018 - current) develops immunotherapies via protein design technologies.
- Lyell ([www.lyell.com](http://www.lyell.com), Seattle, WA, 2018 - current) develops cell-based immunotherapies for treating cancer.
- Dualogics (<https://www.dualogics.com/>, Chapel Hill, NC, 2015 - current) implements an OrthoMab bispecific antibody platform borne from Rosetta design simulations[6,7]
- Digestiva (<https://www.digestiva.net/>; Davis, CA, 2018 - current) computationally engineers enzymes to develop customized proteases and protease enabled products.
- Sana Biotechnology (<http://sana.com/>; San Francisco, CA / Seattle, WA / Boston, MA, 2018 - current) developing therapeutics from engineered cells.
