# Supplementary material for: Better together: Elements of successful scientific software development in a distributed collaborative community
Source: PLoS Comput Biol. 2020 May 4;16(5):e1007507. doi: 10.1371/journal.pcbi.1007507 (PMC7197760; doi:10.1371/journal.pcbi.1007507)
Supplement: S7 Text — (DOCX) [file pcbi.1007507.s008.docx]

## S7 Text: Details of Remaining Collective Challenges

### Technical Challenges

(1) **Maintainability and usability**: Scientific software is often developed by scientists with no training in computer science or software engineering, which complicates balancing “good” code design, performance and documentation with achieving some scientific goal; the dearth of incentives for academics to write well-designed, usable, maintainable code only exacerbates this tension. We addressed this challenge by creating workflows that incorporate writing documentation into the development process (*e.g.* writing pseudo-code as comments and adding the code after, encouraging developers to write documentation while code is compiling), training developers, establishing publication standards that require demos or protocol captures for scientific reproducibility, and enacting requirements for merging code into the main development branch. For the latter we use the GitHub pull-request system with required code reviews, which has served our community extremely well. While this can create a barrier to contributing code, the quality of the contributed code is instantly raised to maintain a balance between quality versus quantity: *Code is cheap but good code is expensive*. These solutions were determined collaboratively from the bottom up, which is one of the reasons graduate students and postdocs continue to be very committed to Rosetta development and its community.

(2) **Documentation**: The lack of helpful documentation for most scientific software tools is mostly due to the incentive structure in academic settings. The existence and quality of documentation for Rosetta has drastically improved over the past decade, but there is still room for improvement. We found that documentation can be enhanced by using an easily editable format (such as the Gollum wiki[8]), publication standards (if a tool is published and merged into the master branch, documentation is required), XRW hackathons (we had two one-week hackathons solely dedicated to writing and improvement of documentation and demos), and efficient workflows. We also debated hiring dedicated personnel to write documentation. However, we recognized that writing documentation for complex scientific software tools is challenging to outsource because the scientific expertise and detailed understanding of the tool required to write useful documentation is held by the developers themselves.

(3) **Code and protocol duplication**: The Rosetta codebase as of 2019 contains about 3.1 million lines of code. Although Rosetta can solve a variety of complex problems at the interface between biology, chemistry, physics and mathematics, we estimate that the codebase could be reduced by half without a significant loss of functionality. Codebases can be cleaned with the aid of specialized tools that identify duplicated code, but a better strategy is to avoid the problem *a priori* by training developers. A much more pervasive and complex issue (especially in Rosetta), is protocol duplication; meaning that the basic steps of a particular protocol are duplicated while the code differs on a line-by-line basis. Protocol duplication arises from poor discoverability of existing protocols, which could be improved by prohibiting acronyms or abbreviations (that often only the developer knows) and setting up naming conventions for protocols, files and functions early on. Also, many implementations are insufficiently general to be easily extended or used in a slightly different way, again exacerbated by code development by non-computer scientists. It can be easier to write new code than to read, understand and edit or generalize someone else’s code. We also found that simple tasks are more often duplicated than complex protocols, which could be prevented by better communication and early pull-request reviews.

(4) **In-house developed features**: Rosetta has a number of in-house developed components, either because of lack of certain tools or libraries, insufficient flexibility of such tools or libraries, or there were other concerns about their implementation or licenses. As an example, we implemented several minimizers and K-means clustering algorithms, a random number generator, a channel-based logging system, our own command-line options system, most of our testing framework, job and task distribution systems, and source code linting tools such as the beautifier. We also customized the SCons build system to suit our needs. While Rosetta’s development began before many of the tools and libraries were available that exist nowadays, leading us to implement our own solutions, we now must maintain and support those or undertake refactoring efforts to deprecate our in-house solution in favor of a standard solution. For other scientific software projects that started later or have a smaller codebase, we highly encourage the use of established tools.

(5) **Input / Output**: The input/output (I/O) handling in Rosetta is heterogeneous and could be improved; however, incentives for harmonizing these components are lacking or insufficient. We support I/O for three different database types: SQLite, MySQL, and Postgres, as this depends on the usage at various institutions. Rosetta maintains several dozen custom file formats for data required for specific protocols, in addition to an entire system (“silent files”) for efficiently storing protein conformations that is only operable within Rosetta: this complexity could be reduced. Accordingly, code for parsing these file formats is required and needs to be maintained. Rosetta lacks standardization in error-handling or parsing, and our command line interface currently supports about 4,400 options (partly due to namespace multiplicity). These challenges could be mitigated by choosing specific file formats early, as well as through creating coding conventions, encouraging communication between developers, and providing incentives for streamlining IO.

(6) **Software stability versus cutting-edge development**: Developers need to recognize the inherent conflict between state-of-the-art code that evolves through refactoring or addition/modification of features and mature code that remains stable and maintains its API, especially across interfaces. One example has become apparent in our community recently: as the PyRosetta API mirrors the C++ code, changes at the C++ level are passed on when that revision is used to build PyRosetta. Sometimes these changes can result in broken PyRosetta scripts that leave users frustrated and confused.

(7) **Interfaces to the codebase**: Rosetta has various interfaces to its functions – command line, PyRosetta, RosettaScripts, Foldit, InteractiveRosetta – each with their own communities and tradeoffs for efficiency, ease-of-access, and flexibility. While this can be useful for developers with different skill levels, there is no supervision encouraging us to focus on a single interface and continuously improve it. Ultimately, every interface must be maintained (possibly indefinitely) – adding new interfaces should therefore be done thoughtfully.

(8) **Legacy code**: Rosetta still contains machine-translated Fortran legacy code in low-level libraries from 15 years ago, which is difficult to read, maintain, or replace. Similarly, there are large chunks of historical code that are either still used or difficult to remove or update because the implementation has tightly coupled building blocks at a low level. Identifying legacy code is a challenge in itself. In a codebase like Rosetta this could require collecting user-data to identify which protocols are still used. This information is difficult to obtain, especially from commercial users.

(9) **Parallelization for GPUs**: It is intuitive for a biochemist to organize vectors of objects that can be manipulated. This choice facilitates development but is inefficient for performance, especially in a massive codebase. A more efficient approach would be to create objects of vectors, operations of which could be much more efficiently handled and parallelized for Graphics Processing Units (GPUs). An example is the scoring machinery in Rosetta, which could be reoptimized for runtime on GPU. While efforts to implement parts of the scoring machinery for GPU architectures are currently underway, it remains difficult to adapt Rosetta to massively parallel compute models due to its size. Further, the size of the codebase and developer community create major obstacles to a complete re-write as was done for Rosetta3 about 12 years ago. Since the large size of the codebase is partially caused by code and protocol duplication and limited use of third-party libraries, these earlier mistakes now create major challenges for adaptation to GPUs.

(10) **Development environments**: The development environments that are used in the Rosetta community vary by developer preference and laboratory. For instance, some laboratories use GNU/Linux while others develop on macOS, with or without shared filesystems. The Xcode Interactive Development Environment (IDE) only runs on macOS, others use Eclipse, Visual Studio, or don’t use an IDE at all. Six different build systems are supported, but the primary tool is SCons, which is highly customizable but can be slow. The SCons build files are used to generate inputs for cmake/make, cmake/ninja, XCode, Qt, and Visual Studio. While flexibility can be useful, it creates challenges for new developers without development background to master not only the highly complex codebase and various scientific subject areas, but also the range of tools available for development.

### Social Challenges

(11) **Inclusion of new PIs**: New institutions are currently joining the RosettaCommons at a rate of six per year. With over 50 active PIs, including new PIs into the RosettaCommons is a current open question. Presently, prospective new PIs need their institution to sign the participation agreement to join the RosettaCommons, a nomination letter from a current PI, and a majority vote from the executive committee. This approach favors membership to those already intimately connected within the community and disfavors outside scientists. How do we ensure equitable access to the community while also ensuring continuation of our values of open collaboration and community culture? Who can join, when? How much do they need to be connected to the current PIs?

**(12) Maintaining a small-community feel**: Our conferences used to be small, and all attendees were personally introduced and we each became familiar with almost all other attendees. At 270 participants, RosettaCon is now larger than the venue can handle which precludes meeting everyone, let alone forging deep connections with all attendees. To forge cross-lab connections, we now hold break-out groups organized by topic or with random matchings, and have mentoring, networking, and social events (hikes or games). In the past few years, many people who wished to attend were unable to because of space constraints. The problem is compounded by additional interest from industry and from new labs currently unaffiliated with the RosettaCommons. We are debating to move to a larger venue or break the meeting into multiple meetings with smaller groups. Our hope is to maintain our community-focused culture even as the group grows to the next stage.

(13) **Increasing diversity, equity and inclusion**: Social justice struggles progress over timescales of decades and longer. While we already have evidence of the impact of our efforts in inclusion and diversity, the majority of our PIs is still predominantly white men, and it takes time for trainees to move through multiple career stages to become PIs. In the meantime, we are committed to long-term work to include scientists along different dimensions of identities at all professional stages in our community.

(14) **Credit and authorship:** Advances in Rosetta stand on the collective work of hundreds of scientists over the past twenty years. New developers are not always aware of the origin of each part of Rosetta that they use in their work, making it difficult to cite the proper set of publications. This is our first manuscript listing the Consortium as an author; we are debating whether and how it would be appropriate to acknowledge credit in this mechanism for scientific papers. In other large-scale science projects, for instance in astronomy or genomics, hundreds of authors can be listed, acknowledging those with deep technical contributions in building and maintaining the toolset. We are considering such a model to better recognize the contributions of the core code creators.

### Licensing and Dissemination Challenges

(15) **Inclusion of industry contributions:** When developers leave academia and join industry, they are restricted to the commercial release version of Rosetta and are no longer permitted to contribute code back to our main codebase; that is, they no longer have access to the active, development branches of Rosetta. However, as more of our trainees and developers move to industry, more code development is being done by scientists in industry, and there currently is no mechanism for them to contribute back to the RosettaCommons codebase. Issues of ownership, intellectual property, and supervision are to be considered. A committee of Rosetta board members and industry representatives are currently debating these issues and creating a proposal to include industry developers in the RosettaCommons.

# References:

1. CxxTest. http://cxxtest.com/.

2. Ó Conchúir S, Barlow KA, Pache RA, Ollikainen N, Kundert K, O’Meara MJ, et al. A Web Resource for Standardized Benchmark Datasets, Metrics, and Rosetta Protocols for Macromolecular Modeling and Design. Zhang Y, editor. PLoS One. 2015;10: e0130433. doi:10.1371/journal.pone.0130433

3. Valgrind. http://www.valgrind.org/.

4. Gordon SR, Stanley EJ, Wolf S, Toland A, Wu SJ, Hadidi D, et al. Computational Design of an α-Gliadin Peptidase. J Am Chem Soc. 2012;134: 20513–20520. doi:10.1021/ja3094795

5. Wolf C, Siegel JB, Tinberg C, Camarca A, Gianfrani C, Paski S, et al. Engineering of Kuma030: A Gliadin Peptidase That Rapidly Degrades Immunogenic Gliadin Peptides in Gastric Conditions. J Am Chem Soc. 2015;137: 13106–13. doi:10.1021/jacs.5b08325

6. Lewis SM, Wu X, Pustilnik A, Sereno A, Huang F, Rick HL, et al. Generation of bispecific IgG antibodies by structure-based design of an orthogonal Fab interface. Nat Biotechnol. 2014;32: 191–8. doi:10.1038/nbt.2797

7. Leaver-Fay A, Froning KJ, Atwell S, Aldaz H, Pustilnik A, Lu F, et al. Computationally Designed Bispecific Antibodies using Negative State Repertoires. Structure. 2016;24: 641–651. doi:10.1016/j.str.2016.02.013

8. Gollum wiki. In: https://github.com/gollum/gollum.
