# Supplementary material for: Better together: Elements of successful scientific software development in a distributed collaborative community
Source: PLoS Comput Biol. 2020 May 4;16(5):e1007507. doi: 10.1371/journal.pcbi.1007507 (PMC7197760; doi:10.1371/journal.pcbi.1007507)
Supplement: S1 List — (DOCX) [file pcbi.1007507.s009.docx]

S1 List: RosettaCommons Consortium Members

People listed below are active members of the RosettaCommons as of the time of writing of this manuscript. * indicates senior developers (other than PIs) who have been involved in the RosettaCommons for seven or more years (usually with affiliations in multiple Rosetta labs).

# Arizona State University

Jeremy Mills, Principal Investigator

Patrick R. Gleason

Bethany Kolbaba Kartchner

Patrick I. Kelly

# Australian National University

Thomas Huber, RosettaCommons Affiliate

Kala Bharath Pilla

# Cornell University

Elizabeth Kellogg, Principal Investigator

# École Polytechnique Fédérale de Lausanne (EPFL)

Bruno E. Correia, Principal Investigator

Jaume Bonet

Zander Harteveld

Andreas Scheck

Patrick Barth, Principal Investigator

Andreas Fuglistaler

Rajendra Sharma

# ETH Zurich

Jacob Corn, Principal Investigator

# Flatiron Institute & New York University

Richard Bonneau, Principal Investigator, RosettaCommons Web and Social Media Chair

Daniel Berenberg

James Eastwood

Julia Koehler Leman*, Diversity Committee member

Vikram K. Mulligan*

P. Douglas Renfrew*, Diversity Committee member

# Fox Chase Cancer Center

Roland Dunbrack, Principal Investigator

Bulat Faezov

Simon Kelow

Vivek Modi

Maxim Shapovalov

Qifang Xu

John Karanicolas, Principal Investigator, Executive Board Member-At-Large

Karen Khar (University of Kansas)

#

# Fred Hutchinson Cancer Center

Phil Bradley, Principal Investigator

# Graz University of Technology

Gustav Oberdorfer, Principal Investigator

Wael Elaily

Julia Messenlehner

Adrian Tripp

# Harvard University

Sergey Ovchinnikov, Principal Investigator

Haobo Wang

# Hebrew University

Ora Schueler-Furman, Principal Investigator, Diversity Committee member

Ziv Ben Aharon

Nawsad Alam

Oriel Goldstein

Alisa Khramushin

Orly Marcu

Barak Raveh

Tomer Tsaban

# Hubei University of Technology

Sen Liu, Principal Investigator

# Institute of Protein Innovation (Boston)

Chris Bahl, Principal Investigator, Diversity Committee member

Samaneh Mesbahi-Vasey

Ian Truebridge

# Johns Hopkins University

Jeffrey J. Gray, Principal Investigator, RosettaCommons Diversity Chair,
Director of Rosetta Summer Intern Program

Rebecca F. Alford*, Diversity Committee member

Ameya Harmalkar

Jeliazko R. Jeliazkov

Sergey Lyskov,* RosettaCommons Software Engineer

Sai Pooja Mahajan

Nicholas Marze

Camille Mathis, Program Coordinator of Rosetta Summer Intern Program

Matt Mulqueen, RosettaCommons Systems Administrator

Morgan L. Nance

Shourya S. Roy Burman

Sudhanshu Shanker

Jing Zhou

Jason W. Labonte, Principal Investigator (visiting appointment at Franklin & Marshall College)

# Linnaeus University

Sinisa Bjelic, Principal Investigator

# Los Alamos National Laboratory

Charlie E. M. Strauss, Principal Investigator, Executive Board Member-At-Large

Ramesh K. Jha, Principal investigator

Jacob C. Minor

# Lund University

Ingemar André, Principal Investigator

Antonius G.L. Hoevenaars

Mads Jeppesen

Wojciech Potrzebowski

# National Renewable Energy Laboratory

Deanne Sammond, Principal Investigator, RosettaCommons Treasurer

# Northeastern University

Seth Cooper, Principal Investigator

Lorna J. L. Dsilva

Matthew Hantsbarger

Robert Kleffner

Josh Aaron Miller

Uttkarsh Narayan

# Northwestern University

Gabriel Rocklin, Principal Investigator

# Ohio State University

Steffen Lindert, Principal Investigator

Melanie Aprahamian

Justin Seffernick

# Peking University

Chu Wang, Principal Investigator

Yuan Liu*

# Rensselaer Polytechnic Institute

Chris Bystroff, Principal Investigator

Benjamin Walcott

# Rutgers University

Sagar Khare, Principal Investigator

Kristin Blacklock

Elliott Dolan

William Hansen

Changpeng Lu

Joseph H. Lubin, Diversity Committee member

Aliza Rubenstein

Maria Szegedy

Brahm Jonathan Yachnin

Manasi Pethe, Diversity Committee member

# Scripps Research Institute

William Schief, Principal Investigator

Jared Adolf-Bryfogle

Xiaozhen Hu

Sebastian Rämisch

# Stanford University

Po-ssu Huang, Principal Investigator, 2019 Rosetta Conference co-Chair

Namrata Anand

Raphael Eguchi

Rhiju Das, Principal Investigator

Caleb Geniesse

Kalli Kappel

Ramya Rangan

Andrew Watkins*

# The Wistar Institute

Daniel W. Kulp, Principal Investigator

# University of Bristol

Fabio Parmeggiani, Principal Investigator

# University of California, Davis

Justin Bloomfield Siegel, Principal Investigator, RosettaCommons Business Development Chair

Simon Kit SangChu

Youtian Cui

Jason Fell

Wai Shun Mak

Yue Zhang

Stephanie Contreras

Ashley Vater

Peishan Huang

Katherine Buse

Melissa Wills

Colin Milburn

Ranjodh Singh Dhaliwal

Vladimir Yarov-Yarovoy, Principal Investigator

Aiyana M. Emigh

Brandon J. Harris

Ian H. Kimball

Jan Maly

Phuong Tran Nguyen

# University of California, Los Angeles

Lin Jiang, Principal Investigator

Kevin A. Murray

Woo Shik Shin

#

# University of California, San Francisco

Tanja Kortemme, Principal Investigator, RosettaCommons Grants Co-Chair

Kyle Barlow

Anum Glasgow

Amanda Loshbaugh

James Lucas

Shane Ó Conchúir

Noah Ollikainen

Xingjie Pan

Amelie Stein

# University of California, Santa Cruz

Nikolaos G. Sgourakis, Principal Investigator

Andrew C. McShan

Santrupti Nerli

# University of Colorado

Timothy Whitehead, Principal Investigator, RosettaCommons Membership Chair

Paul Steiner

# University of Denver

Scott Horowitz, RosettaCommons Affiliate

# University of Georgia

Eva-Maria Strauch, Principal Investigator

Karen Juliana Gonzalez Restrepo

Raulia Syrlybaeva

Issac Torres

# University of Illinois Urbana-Champaign

Erik Procko, Principal Investigator

# University of Kansas

Andrea Bazzoli

David Johnson

# University of Maryland

Brian G. Pierce, Principal Investigator

Ragul Gowthaman

Johnathan D. Guest

# University of Massachusetts, Dartmouth

Firas Khatib, Principal Investigator

# University of Michigan, Ann Arbor

Matthew J. O'Meara, Rosetta Affiliate

# University of North Carolina

Brian Kuhlman, Principal Investigator, RosettaCommons Awards Chair

Matthew Cummins

Odessa Goudy

Sharon Guffy

Stephan Kudlacek

Andrew Leaver-Fay*

Jack Maguire

Thanh Phan

Frank Teets

David Thieker

Hayretin Yumerfendi

# University of Tokyo

Daisuke Kuroda, Rosetta affiliate

# University of Warsaw

Dominik Gront, Principal Investigator, 2019 Rosetta Conference Co-Chair

Justyna D Kryś

Joanna M Macnar

# University of Washington

David Baker, Principal Investigator, RosettaCommons Director

Benjamin Basanta

Ariel Ben-Sasson

Stephanie Berger

Sherry Bermeo

Matthew Bick

Scott Boyken

Jilliane Bruffey

TJ Brunette

Cassie Bryan

Gabe Butterfield

Ralph A. Cacho

Shane Caldwell, Diversity Committee member

Longxing Cao

Zibo Chen

Tamuka Chidyausiku

Qian Cong

Alexis Courbet

Brian Coventry

Fatima A. Davila H.

Jiayi Dou

Nathan Ennist

Ali Etemadi

Jorge Fallas

Jeff Flatten

Alex Ford

Stacey Gerben

Lukasz Goldschmidt

Inna Goreshnik

Ian Haydon, Diversity Committee member

Derrick R. Hicks

Parisa Hosseinzadeh, Diversity Committee member

Yang Hsia

Indrek Kalvet

Christine Kang, Diversity Committee member

Ryan Kibler

David Kim

Indigo King

Jason C. Klima

Brian Koepnick

Elif Nihal Korkmaz

David La

Marc LaJoie

Robert Langan

Gyu Rie Lee

Yu-Ru Lin

Thomas Linsky

Sidney Lisanza

Ajasja Ljubetič

Enrique Marcos

Sinduja K. Marx

Michelle Matsunaga

Rubul Mout, Diversity Committee member

Adam Moyer

Una Nattermann, Diversity Committee member

Jorgen Nelson

Christoffer Norn

Hahnbeom Park

Harley Pyles

Anindya Roy

Franziska Seeger

Will Sheffler

Hao Shen

Daniel-Adriano Silva

Yifan Song

Summer Thyme

George Ueda

Umut Ulge

Anastassia Vorobieva

Kathy Wei

Chunfu Xu

Ta-Yi Yu

Dmitri Zorine

Frank DiMaio, Principal Investigator

Carson Adams

Daniel Farrell

Brandon Frenz

Ryan Pavlovicz

Gabriella Reggiano

Ray Yu-Ruei Wang

Guangfeng Zhou

Dennis Hanson, Co-Motion Rosetta Licensing Manager

Neil King, Principal Investigator

Annie Dosey

Quinton Dowling

Dan Ellis

Karla-Luise Herpoldt, Diversity Committee member

Jing Yang Wang

# University of Wisconsin–Madison

Srivatsan Raman, Principal Investigator

Anthony Meger

Nicholas Hoppe

# University of Zurich

Lars Malmström, Principal Investigator, Executive Board Secretary

Hamed Khakzad

# Vanderbilt University

Jens Meiler, Principal Investigator, RosettaCommons Documentation Chair

Diego del Alamo

Brian J. Bender

Benjamin Brown

Nina G. Bozhanova

Amanda Duran

Jessica Finn

Darwin Y. Fu

Alican Gulsevin

Harikrishna Sekar Jayanthan

Georg Kuenze

Brennica Marlow

Rocco Moretti*

Benjamin Mueller

Elleansar Okwei

Michael Pritchard

Amandeep Sangha

Marion Sauer

Clara Schoeder

Samuel Schmitz

Alex Sevy

Jonathan H. Sheehan

Shannon Smith

Oanh Vu

Hope Woods

# Weizmann Institute

Assaf Elazar

Gideon Lapidoth

Jonathan Weinstein

Nir London, Principal Investigator

Barr Tivon

Daniel Zaidman

# Wesleyan University

Colin Smith, Principal Investigator

# Westlake University

Peilong Lu, RosettaCommons Affiliate Member

# *Foldit Players*

Foldit players contribute to the RosettaCommons through their folding and designs of proteins through the game interface, and through algorithmic and score function improvements that have originated from their work.

# *Commercial Partners in the RosettaCommons*

A few companies have people who are also active developers of Rosetta. All code contributions from these individuals become the shared property of the RosettaCommons institutions, which currently includes only non-profit entities.

# Cyrus Biotechnology

Steven Lewis*, Company Liaison

Sam Deluca*

Javier Castellanos

# Lyell

Brian Weitzner*, Company Liaison

# Rosetta Design Group

Xavier Ambroggio, CEO and Company Liaison
